# Supplementary material for: Real-time estimation of phase and amplitude with application to neural data
Source: Sci Rep. 2021 Sep 10;11:18037. doi: 10.1038/s41598-021-97560-5 (PMC8433321; doi:10.1038/s41598-021-97560-5)
Supplement: Supplementary file 1 — Supplementary Information. [file 41598_2021_97560_MOESM1_ESM.pdf]

# Real-time estimation of phase and amplitude with application to neural data. Supplementary Material

**Michael Rosenblum[1,*], Arkady Pikovsky[1], Andrea A. Kühn[2], and Johannes L. Busch[2]**

[1]Department of Physics and Astronomy, University of Potsdam, Karl-Liebknecht-Str. 24/25, D-14476 Potsdam-Golm, Germany
[2]Movement Disorders and Neuromodulation Unit, Department of Neurology, Charité – Universitätsmedizin Berlin, Charitéplatz 1, D-10117 Berlin, Germany
[*]corresponding author: mros@uni-potsdam.de

## ABSTRACT

Here we present the code for the algorithms described in the paper and additional plots.

## Code

The algorithms are implemented in Matlab® and can be found in separate files in this Supplementary Material or downloaded from this site. We provide three files:

- `ModelDataAllAlg.m` contains functions for all three algorithms and reproduces Fig. 1. Additionally, this code plots the instantaneous amplitudes.
- `ResOsz.m` contains code used to process the tremor data and to produce Fig. 3. Additionally to frequency adaptation used in the model data example, this program removes baseline fluctuations.
- `NonResOsz.m` contains code used to process the brain activity data and to produce Fig. 4.

## Estimation error

To quantify the uncertainty in the causal estimation, we use the training interval. For this interval, we compute the circular difference, $\psi$, between the causal phase, $\varphi_N$, and the Hilbert phase, $\varphi_H$. Next, we perform running average for $\psi^2(a_N)$ to obtain an estimate of the variance $\sigma^2(a_N)$. This is illustrated in Fig. 1.

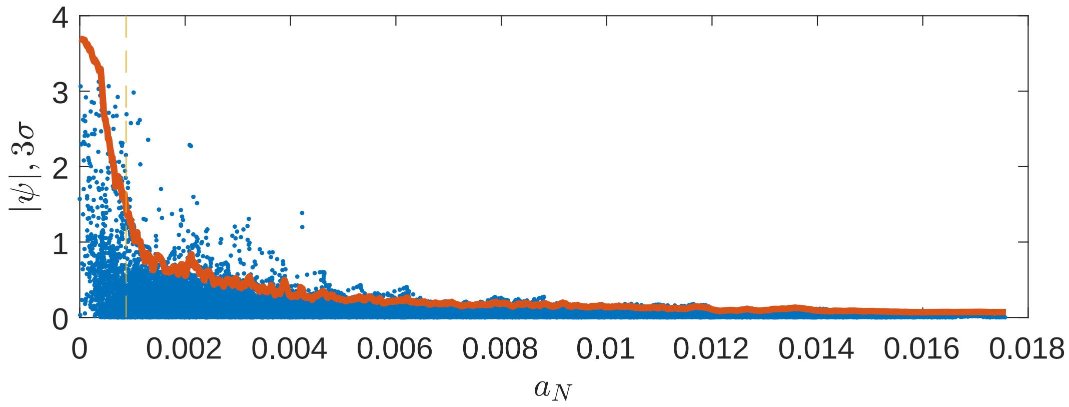


**Figure 1.** Circular differences $\psi$ (blue dots) and estimated standard deviation $\sigma(a_N)$. The red curve corresponding to $3\sigma(a_N)$ represents a good estimation of the confidence interval. The dashed vertical line indicates the threshold $\max(a_N)/20$.

Figure 2 presents the same data in a logarithmic scale. In this representation, the dependence of $\ln(\sigma(a_N))$ on $\ln(a_N)$, for $a_n > \max(a_N)/20$, is nicely fited with a straight line, indicating the power-law dependence $\sigma(a_N) \approx 6 \cdot 10^{-4} \cdot a_N^{-0.94}$. We notice

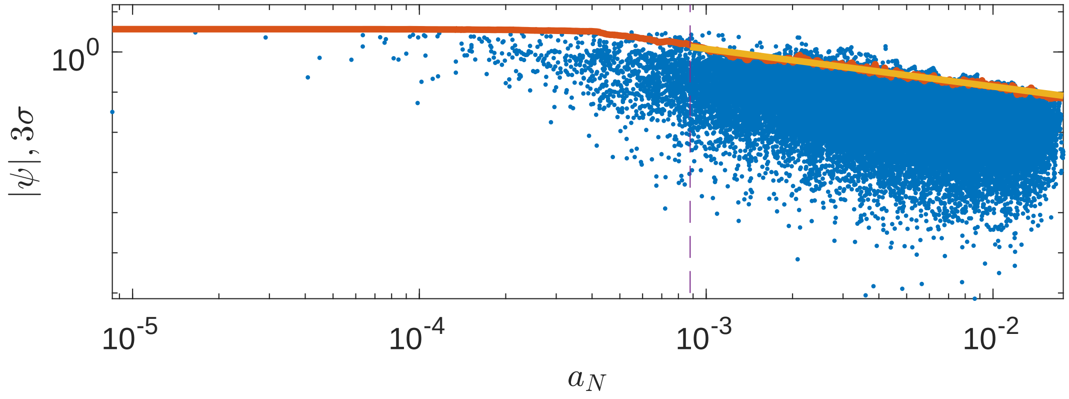


**Figure 2.** The same data as in Fig. 1, but in the logarithmic scale. The dashed vertical line indicates the threshold $\max(a_N)/20$. The yellow line shows the linear fit for the amplitudes above the threshold.

that one can avoid the fitting and use the original function $\sigma(a_N)$, given by its values at many discrete values of its argument (lookup table). For a new value of the argument, the value of the function is obtained via local linear interpolation.

Similarly, we quantify the uncertainty in the amplitude estimation. Performing a running average for $(a_H - a_N)^2$, we obtain $\sigma^2(a_N)$. In contradistinction to the phase estimate, here, the standard deviation $\sigma$ weakly depends on $a_N$ and can be approximated by a constant (see Fig. 3). This observation makes the algorithm extremely simple: we compute the standard deviation of $a_H - a_N$ over the training interval and use this value to provide the error bars for the causal computation.

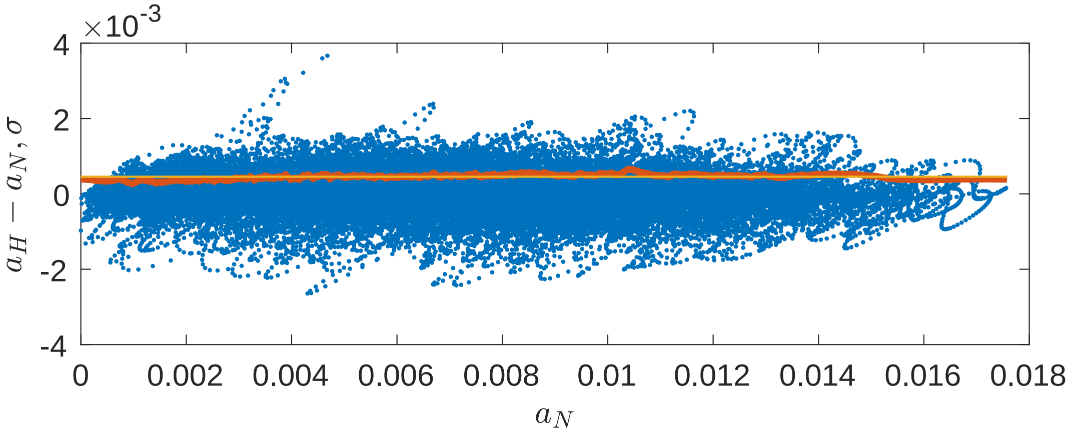


**Figure 3.** The standard deviation for the amplitude error $a_H - a_N$ (red curve) weakly depends on $a_N$ and can be approximated by a constant (yellow line).

## Comparison with other techniques

First, in Fig. 4 we present the results for the technique by Schreglmann et al. Although the technique provides a good estimate for the amplitude, the presion of the phase estimation for this test data is poor. Next, the algorithm is about 30 times slower than our methods.

Next, we present here the results for the technique by Wodeyar et al. The technique fails the test with noisy artificial data (see Eq. (1) in the main text). The results are shown in Fig. 5). We used $10^4$ points out of $10^5$ to train the algorithm. The CPU time for training and processing of remaining points was $\approx 70$ sec. Our algorithms running on the same computer require less than a second.

Furthermore, we compared the performance of the algorithm by Wodeyar et al. and our non-resonant oscillator approach for the analysis of the brain activity. To quantify the performance, we compared mean value $\eta$ and standard deviation $\sigma$ for the circular difference between the non-causal Hilbert phase and the causal one. For our approach we obtained $\eta \approx 0.006$ and $\sigma \approx 0.16$, CPU time about 0.03 sec. The results for the algorithm by Wodeyar et al. are given in Table 1. We see that our approach is more precise and much faster. We emphasize that for computations, we used the Matlab demo function provided

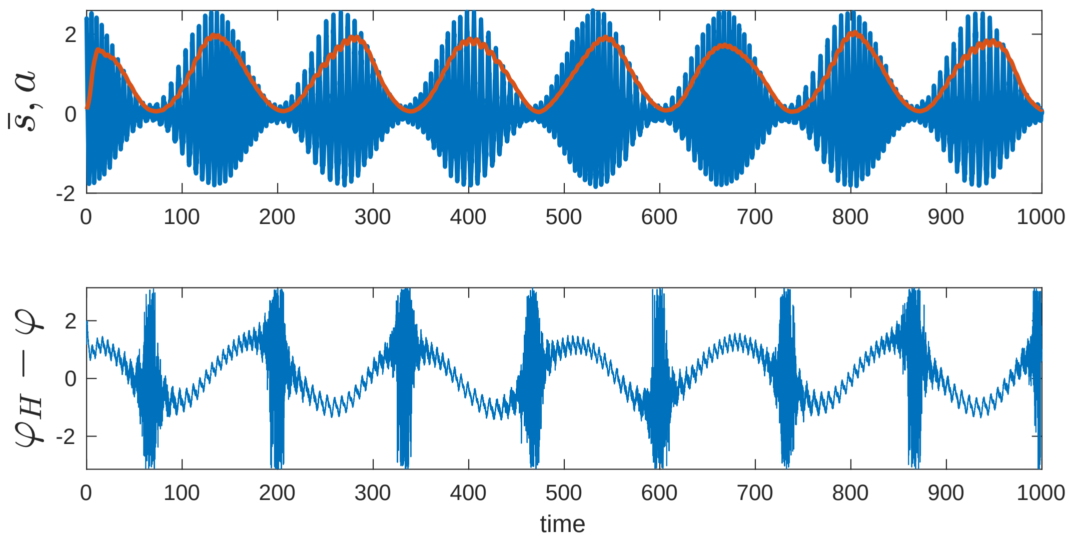


**Figure 4.** A shot epoch of the test signal $\bar{\varepsilon}$ (top panel) and its estimated amplitude (red). Bottom panel shows the difference between the non-causally estimated Hilbert phase and the causal phase. This figure shall be compared with Fig. 2 in the main text.

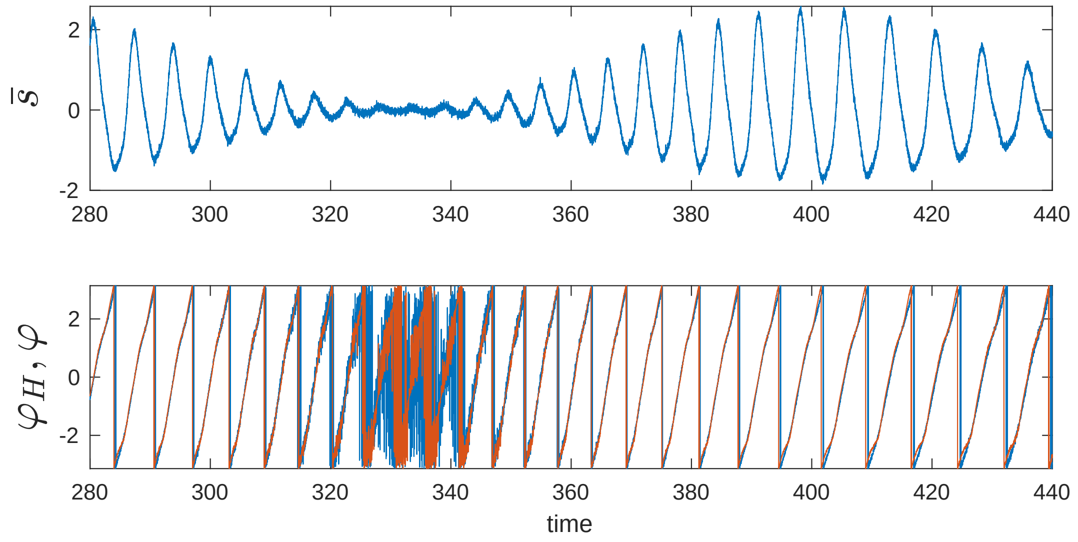


**Figure 5.** A shot epoch of the test signal $\bar{\varepsilon}$ (top panel) and the Hilbert phase (blue) along with the phase obtained by the technique by Wodeyar et al. This figure shall be compared with Fig. 2 in the main text.

by Wodeyar et al. This function does not allow for a proper estimation of the precision. The problem is that the code uses a segment of data to train the algorithm and then processes the rest of the test signal. However, the time required for training is essentially longer than the length of the segment. It means that there is a gap between the segment used for training and the processed data in a real-time application. This gap can cause an additional error if the signal changes its properties with time.

**Table 1.** The results for the algorithm by Wodeyar et al.

| Training interval, sec | CPU time, sec | $\eta$ | $\sigma$ |
|---|---|---|---|
| 5 | 60 | -0.004 | 0.5 |
| 10 | 51 | -0.004 | 0.69 |
| 20 | 44 | -0.026 | 0.97 |
| 30 | 38 | -0.004 | 1.18 |
